# Ultrasound invasion

**DOI:** 10.1186/1824-7288-41-S2-A1

**Published:** 2015-09-30

**Authors:** Rino Agostiniani

**Affiliations:** 1 Department of Pediatrics, Ospedale San Jacopo, Pistoia, Italy

## 

The increasing use of ultrasound in the study of multiple parts of the body, has profoundly changed the diagnostic approach in numerous childhood clinical scenarios.

Some technical aspects, such as minor thicknesses to go through and the lower fat content of tissues, make children optimal subjects for diagnostic ultrasound; exams are also well tolerated by young patients and appreciated by their parents, not painful or invasive and they do not need annoying preparations and/or sedation.

Due to its characteristics ultrasound appears as an ideal integration instrument of the medical examination, increasing accuracy and providing real-time answers to many of the clinical questions open, without interrupting direct communication with the child and his family.

The examination can be performed at the bedside, both in the ward and in the office, but also in emergency situations, in the ED or in the operating room, promptly and with the possibility of seriate checks to appreciate the evolution of the pathology and response to treatment.

The ultrasound fields of application are widening every day: it has always been excellent for soft tissues, and has proved to be useful for the skeleton and lung parenchyma, for long time considered technically not explorable by ultrasound.

The risk is that such and many advantages lead to infinitely expand the indications; in recent years we have seen an exponential growth in demand for ultrasound examinations in pediatric patients, both in primary care and specialized pediatrics.

This situation has caused significant organizational difficulties, due to the limited availability of experienced operators and good quality equipment.

It therefore seems desirable, alongside an increase in the number of operators dedicated to pediatric ultrasound, an intense activity on the issue of the appropriateness of diagnostic tests.

One answer to the problem can be represented by the diffusion of the method in the offices at local level (office ultrasound), for which a number of initiatives aimed at training general practitioners and family pediatricians have already been implemented.

The risk is that the availability of equipment at low cost and the apparent easiness of ultrasound execution technique can encourage a superficial approach to the method, which, in view of its apparent simplicity, remains highly operator-dependent and burdened with a high risk of inaccurate or clearly wrong interpretations.

The definition of well-defined training courses within the Pediatrics specialization school would represent the ideal answer.

